# Studies on the Influence of Host Plants and Effect of Chemical Stimulants on the Feeding Behavior in the Muga Silkworm, *Antheraea assamensis*

**DOI:** 10.1673/031.011.13301

**Published:** 2011-10-04

**Authors:** Kartik Neog, Balagopalan Unni, Giasuddin Ahmed

**Affiliations:** ^1^ Central Muga Eri Research & Training Institute (CMER&TI), Central Silk Board, Lahdoigarh, Jorhat 785 700, Assam, India; ^2^ Biotechnology Division, CSIR-North East institute of Science & Technology, Jorhat 785 006, Assam India; ^3^ Gauhati University, Guwahati, Assam, India

**Keywords:** *Persea bombycina*, flavonoids, preferential feeding, semi-synthetic diet

## Abstract

The feeding habits of *Antheraea assamensis*, Helfer (Lepidoptera: Saturniidae) larvae towards the leaves of its four different host plants, *Persea bombycina* King ex. Hook (Laurales: Lauraceae), *Litsea polhantha* Jussieu, *L. salicifolia* Roxburgh ex. Nees and *L. citrata* Blume, and the chemical basis of feeding preference were investigated. Nutritional superiority of young and medium leaves with respect to soluble protein, total phenol and phenylalanine ammonia lyase activity was observed in the leaves of *P. bombycina* compared to other host plants. Attraction and feeding tests with detached leaves and artificial diet with different chemical stimulants revealed that a mixture of the flavonoids, myrcetin, and 7, 2', 4' trimethoxy dihydroxy flavone with sterol compound β-sitosterol elicited the most biting behavior by *A. assamensis* larvae. While linalyl acetate alone attracted larvae towards the leaves of the host plants, a mixture of caryophyllene, decyl aldehyde and dodecyl aldehyde was found to both attract them to the host leaves and cause biting behavior. Azaindole was found to deter them from the host plants.

## Introduction

Unique and native to the North Eastern region of India, particularly in the Assam province, the muga silkworm, *Antheraea assamensis*, Heifer (Lepidoptera: Saturniidae) is an economically important insect. The silk that is produced by this insect is highly lustrous and is golden yellow or creamy white in color. The filament length of the cocoon is 500–800 m and the denier is approximately 5.5 (Jolly, et al., 1979). *A. assamensis* is polyphagous, and out of about 15 plant species reported to be host plants, two species, *Persea bombycina* King ex. Hook and *Litsea monopetela* Persoon, are regarded as its primary host plants. Two others, *Litsea salicifolia* Roxburgh ex. Nees and *Litsea citrata* Blume, are considered to be secondary host plants. Its other host plants include *Actinodaphnae obovata* Nees (Blume), *A. anquistifolia* (Blume) Nees, *Cinnamomum glaucescans* (Nees) Drury, *C. glanduliferum* (Wallich) Meisner, *Litsea nitida* (Roxburgh) Hooker f., all of which belong to Lauraceae family. Tree species rarely eaten by this insect belonging to other families include, *Michelia champaca* L. (Magnoliaceae) and *Magnolia sphenocarpa* Hooker f. and Thomson, *Celastrus monospermus* Roxburgh (Celastraceae); *Gmelia arborea* Roxburgh (Verbanaceae), *Zanthozylum rhesta* (Roxburgh) D.C. (Rutaceae); *Zizyphus jujuba* (Rhamnaceae) *etc*. (Neog et al. 2006). *A. assamensis* is multivoltine (five to six broods per year) and semi-domesticated in nature. They are reared outdoors on standing trees. At the end of completion of five larval instars, the larvae crawl down, are collected, and allowed to spin cocoons inside a rearing house.

Phytophagous insects show varying degrees of association with host plants, a particular plant species or group of plants on which they feed (food plant range) (Unni et al. 1996). It is a well-known fact that, for an insect with several host plants, a single plant species may not provide the most beneficial conditions during all parts of the life cycle (Reavey and Lawton 1991, Scheirs et al. 2000, Janz 2002). For example, the best host plant for the larva may not be the best site for the egg or for adult feeding. This preference may vary widely among different host plants of the same family or even among different varieties of the same species (Lederhouse et al. 1992, Janz et al. 2001, Percy et al. 2004, Lopez-Vaamonde et al. 2006). In plant-eating insects where larvae develop on a single host, the ovipositional behavior of adult females determines the larval habitat (Gothard et al. 2005). The relative acceptability of host plants for adult oviposition by herbivorous insects is determined by a balance of numerous internal and external stimulants and deterrents (Miller and Strickler 1984, Bossart and Scriber 1999). External factors may include host plant volatiles, surface chemistry, color, texture, and shape (Rausher 1978, Brown et al. 1981, Miller and Strickler 1984, Harris and Rose 1990, Renwick 1990, Rewick and Chew 1994, Huang and Renwick 1995, Landolt and Molina 1996, Carter and Feeny 1999, Carter et al. 1999, Frankfater and Scriber 1999, 2002).

When an insect is choosing a host-plant, it may use a variety of senses, including smell, sight, touch and taste (Bernays and Chapman 1994). In the first stages of selection, smell and sight are the most important senses because they normally operate at long distances. After the insect lands on a potentially suitable host-plant, touch and taste become more important.

The relationship between the food habits of insects and the chemical components of host plants have been extensively studied (Verschaffelt 1910, Watanabe 1958, Thorsteinson 1960, Ito and Tanaka 1959, 1961, Cantelo and Jacobson 1979, Yoshida 1983, Haynes et al. 1991, Lopez et al. 2000). Host plant selection behavior or feeding preferences are largely mediated by the presence and distribution of secondary metabolites in plants (Frankael, 1959, Lin et al., 1998). These chemicals are classified according to their effect on insect behavior and host-plant selection by insects. Definitions given by Dethier (1960) that are still in use are: attractant, a chemical (volatile) that causes an insect to make orientated movements towards the source of stimulus (plant); repellent, a chemical (volatile) that causes an insect to make orientated movements away from the source (plant) feeding or oviposition; stimulant, a chemical that elicits feeding or oviposition (plant surface compounds); deterrent, a chemical that inhibits feeding or oviposition (plant surface compounds and plant tissues). Flavonoids and related phenolic compounds act as strong feeding deterrents to many insects, but they may act as stimulants for others. (Neilson et al. 1979, McFarlane and Distler 1982, Simmonds 2001, Green et al. 2003). Moreover, chemical stimulants acting at a certain concentration at one receptor of the insect may influence acceptance of the host, while a different concentration of the chemical may cause the insect's central neuron to reject it (Ananthakrishnan, 1991). Choudhury et al. (2006) detected an indole compound azaindole in the leaves of *Persea bombycina* plants that were not preferred by *A. assamensis* larvae and reported that feeding on leaves with high azaindole content (0.3–0.5%) produced flimsy cocoons, fewer number of eggs per laying, and an uneven duration of hatching. Even within Lauraceae-specialized butterflies, there are many important differences between plant toxicity of *P. bombycina* (compared to other Lauraceae species) and different insect species and even populations within the same species (Nitao et al. 1991, Lederhouse et al. 1992). Many specialists on the angiosperms Rutaceae, Magnoliaceae, or Monimiaceae maintained the ability to detoxify the Lauraceae (Scriber et al. 2008, Mercader and Scriber 2008), suggesting that even after millions of years of specialization, behavioral and detoxification abilities still exist for plants that have never been encountered.

There are very few studies on the feeding behavior of *A. assamensis* with respect to chemical stimuli of its host plants. Hazarika (1994) categorized *Machilus* (=*Persea*) *bombycina* idiotypes into most preferred, moderately preferred and least preferred types and suggested that dodecanal and caryophyllene, present dominantly in the most preferred idiotypes, may play the role of olfactory attractants, enhancing the feeding rate of muga silkworms on these plants. In the present report, we studied the behavior of *A. assamensis* larvae towards the leaves of different host plants at different maturity levels and also towards different chemicals reported to have stimulatory effects on insects, including *B. mori* (Ishikawa et al. 1969, Hamamura et al. 1962). Moreover, an attempt was made to induce feeding on an artificial diet fortified with the chemicals by the early instar larvae to test their efficacy.

## Materials and Methods

The experimental materials in the present investigation consist of the muga silkworm *A. assamensis* and its four food plants, *Persea bombycina* King ex. Hook (Laurales: Lauraceae), *Litsea polyantha* Jussieu.*L*. *salicifolia* Roxburgh ex. Nees and *L. citrata* Blume. Disease-free *A. assamensis* eggs were obtained from the Seed Technology Laboratory of Central Muga Eri Research & Training Institute (CMER&TI), Central Silk Board, Govt, of India, Lahdoigarh, Jorhat, India. The host plants were maintained in the experimental field of CMER&TI and the silkworms were reared in the experimental field in outdoor conditions following standard procedures (Chakravorty et al. 2005).

### Insect behavior in response to leaves of different host plants

Based on morphological and physiological characteristics, the leaves of the host plants were identified as tender, medium, and mature. Tender leaves of all the four host plants were morphologically varying in size, pale green, folded, thin, small in size. The weight of the tender leaves ranged from 0.03 g to 0.17 g for *P. bombycina*, 0.06–0.60 g for *L. polyantha*, 0.18–0.51 g for *L. salicifolia* and 0.09–0.19 for *L. citrata*. Medium leaves were unfolded, expanding, deep green in color and weighed from 0.230g to 0.650 g for *P. bombycina*, 0.82–0.96g for *L. polyantha*, 0.80– 0.85 g for *L. salicifolia* and 0.70–0.95 for *L. citrata*. Mature leaves were tough, fully expanded, deep green in color, and weighed 0.03 g to 0.17 g for *P*. *bombycina*, 0.06–0.60 g for *L. polyantha*, 0.18–0.51 g for *L. salicifolia* and 0.09–0.19 for *L. citrata*.

One hundred newly hatched *A. assamensis* larvae were placed at the centre of a circle. *P. bombycina, L. polyantha, L. salicifolia* and *L. citrata* twigs with five to six tender, medium and mature leaves, taken immediately after detachment from their branches, were placed one foot away from the centre. The number of larvae settled on the leaves of the different plants was recorded after 60 minutes and this number is expressed as settling percent.

### Insect *behavior* in response to developmentally different leaves of different host plants

The rearing of *A. assamensis* larvae was conducted to determine growth performance with developmentally different leaves. Three replicates of the tender, semi-mature and mature leaves were maintained. Twenty newly hatched larvae were allowed to feed on different leaf types that were reared up to spinning. The total number of mature larvae that survived out of total larvae brushed during the early stage (1^st^ and 2^nd^ instar), middle stage (3^rd^ and 4^th^ instar) and mature stage (5^th^ instar) was recorded and statistically analyzed.

### Estimation of total phenol and phenylalanine ammonia lyase (PAL) activity

The total phenol content in the leaf samples was estimated by the method described by Malick and Singh (1980). PAL activity in the leaves of different host plants was determined according to the method of Sadasivam and Manickam (2005).

Five hundred milligrams of the leaves were homogenized in 10 mL of a cold 25 m*M* borate-HCl (pH 8.8) buffer containing 5 m*M* β-mercaptoethanol (0.4 mL/L). The homogenate was centrifuged at 12,000 G for 20 minutes and the supernatant was used as a crude enzyme source. PAL activity in crude enzyme was determined by measuring the conversion of L-phenylalanine to transcinnamic acid spectrophotometrically. The reaction mixture contained 0.5 mL borate buffer (pH 8.7), 0.2 mL enzyme solution, 1 mL 0.1M L-phenylalanine and 1.3 mL water. A control was run in which phenylalanine was added after the addition of trichloroacetic (TCA) acid. Following the incubation for 45 minutes at 32° C, an absorbance of 290 nm was measured after the reaction was stopped with the addition of 1M TCA. Enzyme activity was expressed as nano moles cinnamic acid/min/mg protein. A standard curve was prepared by transcinnamic acid. Total protein in the enzyme extract was determined by the method of Lowry et al. (1951).

### Analysis of phenolic compounds

One hundred grams of fresh *P. bombycina* leaves were crushed in the presence of methanol HCl. After crushing into paste, the content was kept under methanol HCl for 48 hours (<) with intermittent shaking. Then the supernatant was collected after filtration followed by centrifugation (5,000 rmp for 10 minutes). The extract was concentrated and dried to a syrupy material that was treated with 2% aqueous NaOH solution and then extracted with 200 mL petroleum ether (40– 60° C). This was further acidified with diluted HCl and extracted with CH_2_Cl_2_. The concentrated organic phase thus obtained was subjected to preparative TLC and compared with standard chemicals for identification.

### Biological evaluation of leaf extracts and chemicals for attraction and biting behavior tests

Eggs of *A. assamensis* or disease-free layings were prepared in the grainage of Central Muga Eri Research & Training Institute, Lahdoigarh, Jorhat, Assam, India. *A. assamensis*,larvae, four to six hours after hatching, with no prior feeding experience, were used for the experiments following the method of Lin et al. (1998) with modifications as described below.

Three pairs of Petri dishes for each of the host plants or standard chemicals were taken and wax coated. Two layers of paper towels and one layer of filter paper (Whatman No. 1) were placed on the dish. The filter papers were soaked with deionized water, which was used to keep the humidity high in each dish. Half pieces of Whatman No. 1 filter paper, 42.5 mm in diameter, were fixed with insect pins for each dish. The discs were then treated with the extracted chemicals or with standard reference chemicals; two discs were treated with solvent control. Ten larvae were placed in the dishes and the number of larvae attracted to or showing biting behavior towards a particular treatment was recorded and expressed as a percent. When larvae showed attraction to or biting behavior towards a particular treatment, their response was considered as positive (+ve), the reverse as negative response (-ve). When it was about 80 percent or more, the response was denoted as highly +ve. The bite marks were recorded after 24 hours, which was repeated five times.

### Experiment on feeding with semi-synthetic diet

To test and confirm the effects of the chemicals used for attraction and biting behavior tests, and also for the beginning of feeding with an artificial or semi-synthetic diet, a basic diet was formulated. Several other diet formulations were prepared with the addition of the chemicals showing positive responses in the previous experiments, singly or in combination as shown in Table 1. Six Petri plates were arranged for each diet as in the above experiment. The diets were placed in the plates and five newly hatched larvae were put on top of the diet inside the Petri plates. Their survivability was recorded up to 2^nd^ instar.

### Preparation of the diet

The dry ingredients were weighed, blended thoroughly and mixed with water. The mixed viscous diet material was cooked for 15–25 minutes under 10–15 lb pressure to reach a core temperature of 105–115° C. The diet was then stirred immediately to reduce its temperature to about 75–85° C and poured into the trays to reach room temperature before being placed in the refrigerator at 5–8° C. This diet can be preserved at 5–10° C for 20–25 days.

### Rearing performance of muga silkworm fed with leaves treated with selected chemicals

The chemicals viz. β-sitosterol, myrcetin, 7, 2′, 4′ trimethoxy dihydroxy flavone, caryophyllene, decyl aldehyde, dodecyl aldehyde, azaindole and gallic acid, which were found to exhibit attraction, biting behavior or deterrent effects were sprayed three times on the leaves of some plants grown on cement tubs under a shed house covered with nylon nets. The first spray occurred one day before brushing, the second spray occurred during second *molt* out, and the third occurred during 4^th^ *molt* out. Three tubs were treated by each chemical and 10 larvae were put in. Rearing was conducted till spinning. Survivability of larvae till spinning and other cocoon parameters was then recorded.

## Results

### Insect *behavior* in response to leaves of different host plants

**Figure 1.**
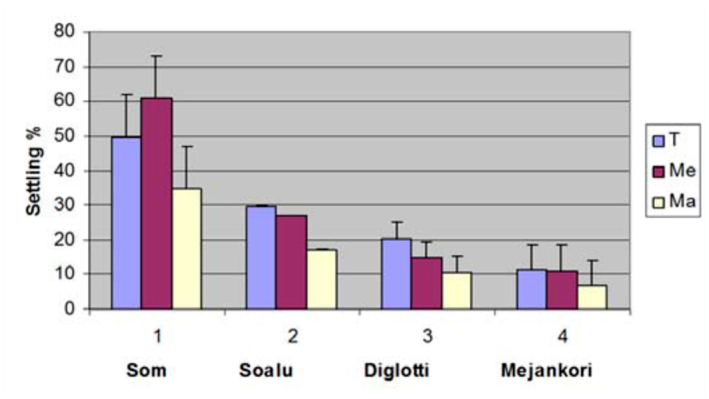
Settling percent of silkworm larvae with respect to tender (T), medium (Me) and mature (Ma) leaves of different host plants, (1) *P. bombycina*, (2) *L*. *polyantha*, (3) *L*. *salicifolia* and (4) *L. citrata*. High quality figures are available online.

Significant variation was observed in the settling percent, i.e., the behavior of the larvae towards leaves of different maturity levels and different host plants. The silkworms were mostly attracted to the medium leaves of *P. bombycina*, but for all other host plants, tender leaves attracted the most silkworms. For settling percent, the differences in means between host plants and maturity level and interaction between host plants and maturity were statistically significant (Figure 1).

### Insect behavior in response to developmentally different leaves of the host plants

**Figure 2.**
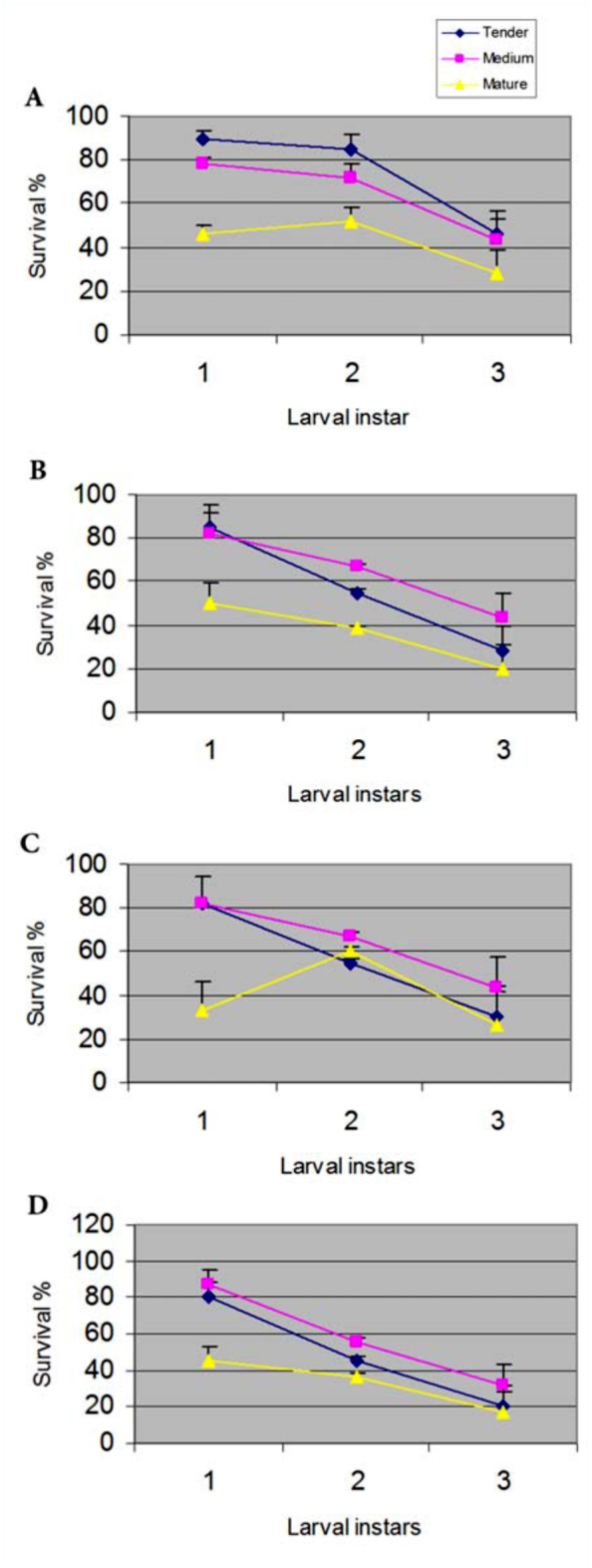
Diagram showing the survival percent of muga silkworms at 1^st^, 1^st^-2^nd^, 2^nd^, 3^rd^-4^th^, 4^th^, and 5^th^ larval instars fed with different leaf types of (a) *P*. *bombycina*, (b) *L*. *polyantha*, (c) *L*. *salicifolia* and (d) *L*. *citrata*. High quality figures are available online.

Significant variation was observed in the survivability of larvae of different instars when they were fed only with tender, medium or mature leaves of different host plants. When the larvae were reared on tender leaves, survival percent of early instar larvae (1–2 instars) was above 80% for all the host plants and 50–70% during 3^rd^ and 4^th^ instars. However, a drastic reduction in survivability was observed during late 5^th^ instar. A higher mortality rate among early instars was observed when the larvae were fed with mature leaves. On the other hand, medium leaves supported the growth of the larvae at a moderate level and resulted in an overall higher survival percent than tender or mature leaves (Figure 2).

### Estimation of total phenol and phenylalanine ammonia lyase (PAL) activity

Significant variation of soluble protein, total phenol, and PAL activity was observed in the leaves of four host plants at three different maturity levels. Tender leaves of *L. polyantha* contained the highest quantity of soluble protein (17.17 mg/g), tender leaves of *P. bombycina* contained the highest total phenol (119.79mg/100g), and mature and medium leaves of *L. citrata* contained the lowest soluble protein (8.86 mg/g), total phenol (15.50 mg/100g). Medium leaves of *P. bombycina* exhibited the highest PAL activity (4.14 nmole/mg/min), while mature leaves of *L. citrata* exhibited the lowest (0.96 nmole/mg/min) (Table 2).

### Analysis of phenolic compounds

From the Methanolic extracts of *P. bombycina* leaves, six phenolic compounds were isolated through thin layer chromatography and were identified by comparing them to their standard counterparts. The relative quantities of these compounds in the leaves of three different maturity levels are presented in Table 3.

### Biological evaluation of chemicals for attraction and biting behavior tests

Muga silkworms showed attraction towards filter papers treated with citral and linalool, but maximum attraction was acheived by linalyl acetate alone, a chemical that attracted more silkworms than a mixture of all three. Caryophyllene, decyl aldehyde, dodecyl aldehyde also showed a highly positive response, but a mixture of all three yielded the highest attraction response (99%). The other chemicals exhibited either deterrent responses (azaindole) or did not have any effect on the muga silkworms. It can be inferred from the experiments that a group of volatile compounds of terpene and aldehyde nature, mainly caryophyllene, decyl aldehyde, dodecyl aldehyde, and linalyl acetate, elicit attraction of muga silkworm towards the leaves of its host plants.

Citral and linalool and linalyl acetate, two chemicals that attracted high numbers of muga silkworms towards the filter paper, did not produce any biting behavior. Beta sitosterol along with myrcetin or 7, 2′, 4′ trimethoxy dihydroxy flavone showed maximum biting behavior by the muga silkworms as revealed by more than 33 bite markings on the papers treated with these chemicals. Individually, these chemicals did not elicit as much biting behavior as they did in combination. Morin and quercetin did not produce as significant a response for muga silkworms. On the other hand, caryophyllene, decyl aldehyde, dodecyl aldehyde, chemicals that produced positive responses on the attraction test, also produced biting behavior. (Table 4).

### Experiment on feeding with semi-synthetic diet

**Figure 3.**
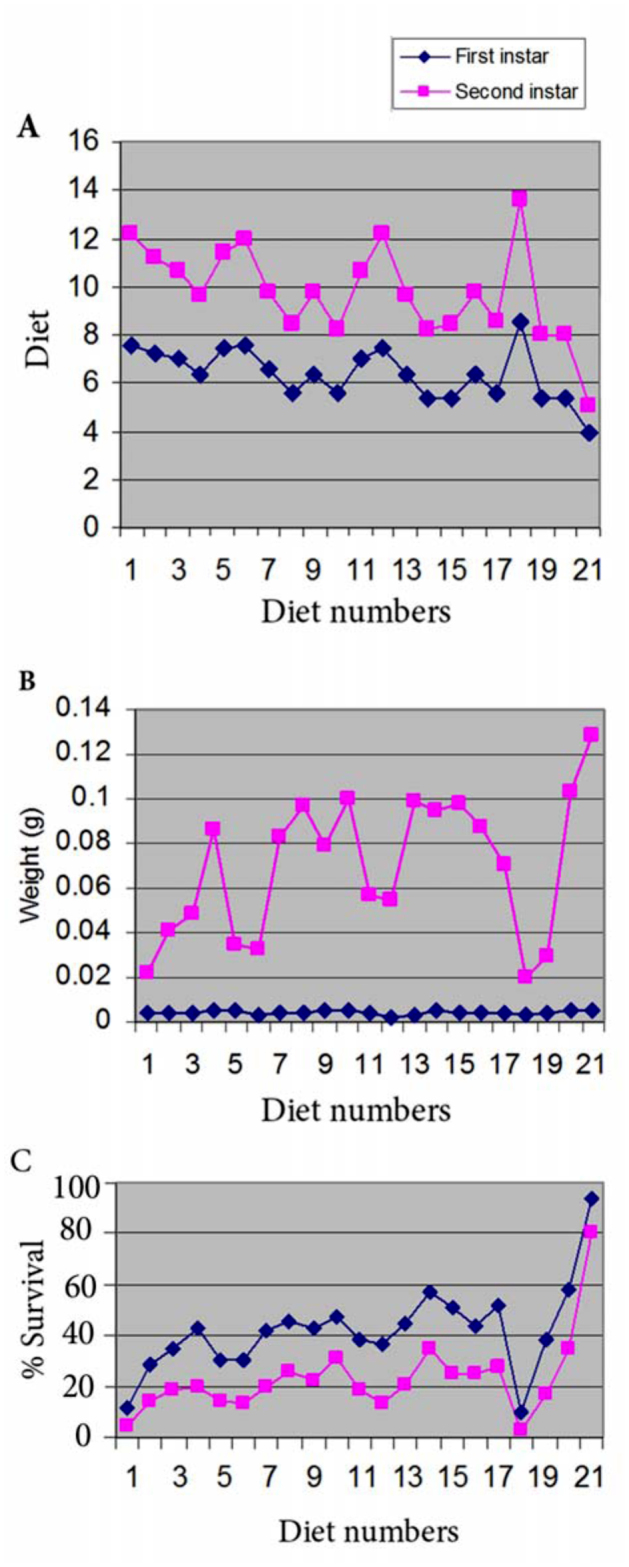
Diagram showing (a) Days required (b) weight in grams and (c) survival percent of first and second instar larvae of muga silkworm; 21=control outdoor rearing. High quality figures are available online.

Molting required the least amount of days (4.5 days) for the 1^st^ instar larvae on diets 14, 15, 16, 19 and 20. For the 2^nd^ instar larvae, molting required the least number of days (8.0 days) for larvae on diets 19 and 20. Larvae fed on diet 18, containing azaindole, experienced the longest larval period for both instars (8.6 days for the 1^st^ and 13.6 days for the 2^nd^ instar). The health of the larvae was very poor and only 3% of them (1 out of 30 larvae) survived up to the 2^nd^ instar. Larvae fed with diet 20 reached the maximum 2^nd^ instar larval weight (0.103 g), followed by those fed with diet 10 (0.100 g). The lowest larval weight was recorded for diet 18 (0.020 g) and diet 1 (0.022 g). Diet numbers 20 and 14 could support almost equally the growth of the larvae up to 2^nd^ instar (35.2% and 34.6% survival percent). The larvae spent more than five days in their 1^st^ instar phase and eight days in the 2^nd^ instar phase. Diet numbers 4, 8, 9, 10, 13, 15, 16, and 17 supported the growth of more than 20% of the larvae up to 2^nd^ instar (Figure 3).

### Rearing performance of muga silkworm fed with leaves treated with selected chemicals

Significant variation in the number of 5th instar larvae harvested, cocoon weight, shell weight and pupal weight was observed when the silkworms were reared on leaves treated with the selected chemicals. The effect on the number of 5th instar larvae was pronounced in the case of leaves treated with myrcetin (55.00%), 7, 2′, 4′ trimethoxy dihydroxy flavone (51.20%), caryophyllene (50.0%), gallic acid (50.0%) and dodecyl aldehyde (48.4%). The other parameters also showed large variations under different treatments. The highest male cocoon weight was recorded from larvae fed on caryophyllene-treated plants, the highest female cocoon and shell weight was measured in β-sitosterol-treated plants. The highest male pupal weight was measured in caryophyllene-treated plants and the highest female pupal weight was measured in dodecyl aldehyde-treated plants (Table 5).

## Discussion

The results of the present study suggest that, out of leaves of different host plants belonging to Lauraceae family, the newly hatched *A. assamensis* were mostly attracted by the medium leaves of *P. bombycina* compared to all other leaves of different age groups. Medium leaves were mostly preferred as revealed by the feeding behavior and survival rate of the larvae during different instars. Visual field observation of the feeding behavior of the larvae also supported this observation. In their study on the Lauraceae specialist insects *Papilio troilus* and *P. palamedes*, Lederhouse et al. (1992) observed highest survival rates and first instar growth rates for *P. troilus* on *Lindera benzoin* and *P. palamedes* on *Persea borbonia*. On their preferred hosts, lifetime growth rates of both specialist insects were much higher than those of related generalist swallowtails. Lederhouse et al. also argued that the restricted geographic range of *P. palamedes* appeared to be the result of oviposition preference rather than larval abilities. Maximum survival and cocoon crop production in *B. mori* was achieved by feeding tender leaves to young larvae (1^st^ -2^nd^ instars), medium leaves to 3^rd^ and 4^th^ instars, and mature leaves to 5^th^ instar larvae (Veda et al. 1997). In the present investigation, it was observed that young larvae fed with mature leaves and vice versa had the lowest survivale rates, suggesting that feeding larvae with leaves of suitable maturity is important for higher survival.

Urzua (2002) reported that the volatile compounds produced by plants can elicit different behaviors in different insect species. For example, camphor acts as a repellent to *Harmonia axyridis*, the multicolored ladybeetle (Coleoptera: Coccinellidae), but is an attractant for *Cicloneda sanguinea* and *Eriopis connexa*. Thorsteinson (1960) confirmed that the mustard oil glycoside, a typical component of cruciferous plants, is responsible for the feeding habits of the larvae of *Euproctis similes*, a species that feeds only on the leaves of the plants belonging to the family Crucifereae.

In the present investigation, a study of the nutritional value of the leaves of each host plant (i.e., soluble protein, total phenol and PAL activity) revealed substantial variation. Young leaves were nutritionally rich compared to mature leaves, as evidenced by higher soluble protein and total phenol content. But the survival percent of *A. assamensis* was higher on medium leaves, a fact that may be due to the greater concentration of defense metabolites found in tender and medium leaves, both of which have high fitness and high probability of attack. PAL activity was significantly higher in medium leaves of *P. bombycina* and *L. polyantha* compared to other leaves. The flavonoids, myrcetin and 7, 2′, 4′ trimethoxy dihydroxy flavone showed higher concentration in tender and medium leaves while others showed higher levels in mature leaves. Presence of higher levels of soluble protein, myrcetin and 7, 2′, 4′ trimethoxy dihydroxy flavone, and PAL enzyme activity can be used as biochemical markers for selecting host plants with leaves of different age groups.

To confirm the role of different phenolic compounds, sterols, and essential oils, attraction, biting and feeding tests were conducted with standard chemicals. These tests indicated that morin and quercetin, that are reported to be factors causing biting behavior of *B. mori*, did not show such response for muga silkworms. Hamamura et al. (1962) classified citral, linalyl acetate, linalool and terpenyl acetate as attractants; sugars, sitosterol, isoquercetin and morin as biting factors; and cellulose, silicate and phosphate as swallowing factors. In the present experiment, a mixture of myrcetin and 7, 2′, 4′ trimethoxy dihydroxy flavone with βsitosterol exhibited the highest biting behavior, β-sitosterol is the active compound of the mulberry plant that induces biting behavior from *B. mori*. In the case of *A. assamensis*, although β-sitosterol's effect on biting behavior is greatly reduced, the compound's effects become pronounced when it is combined with phenolics. The terpinyl compound linalyl acetate alone can elicit attraction of *A. assamensis* larvae, while in case of the *B. mori*, a combination of citral, linalool and linalyl acetate are required for higher attraction behavior. Caryophyllene, decyl aldehyde, dodecyl aldehyde, that showed positive response on the attraction test, also produced biting behavior by the larvae. Feeding of a semi-synthetic diet by first and second larval instars also confirmed the efficiency of these chemicals in effecting attraction, biting behavior and feeding responses by *A. assamensis* larvae. This information may be useful in creating a diet that is capable of supporting the growth of the larvae up to maturity under indoor conditions. Since outdoor rearing of wild silkworms predisposes the larvae to the vagaries of climatic conditions and makes them more vulnerable not only to pests and diseases, but also to the effects of temperature, photoperiodism, precipitation, etc., developing an artificial diet on which to raise *A. assamensis* indoors is of great value.

From the above experiments, it can be inferred that a mixture of phenolic compounds myrcetin, 7, 2′, 4′ trimethoxy dihydroxy flavone with sterol compound β-sitosterol will elicit the greatest biting behavior by *A. assamensis*. While linalyl acetate induces attraction towards leaves of muga host plants, a mixture of caryophyllene, decyl aldehyde and dodecyl aldehyde is the best at inducing attraction of larvae to leaves and induce biting behavior. Thus, the factors responsible for attraction and biting behavior are quite different from that of *B. mori*.

**Table 1.**
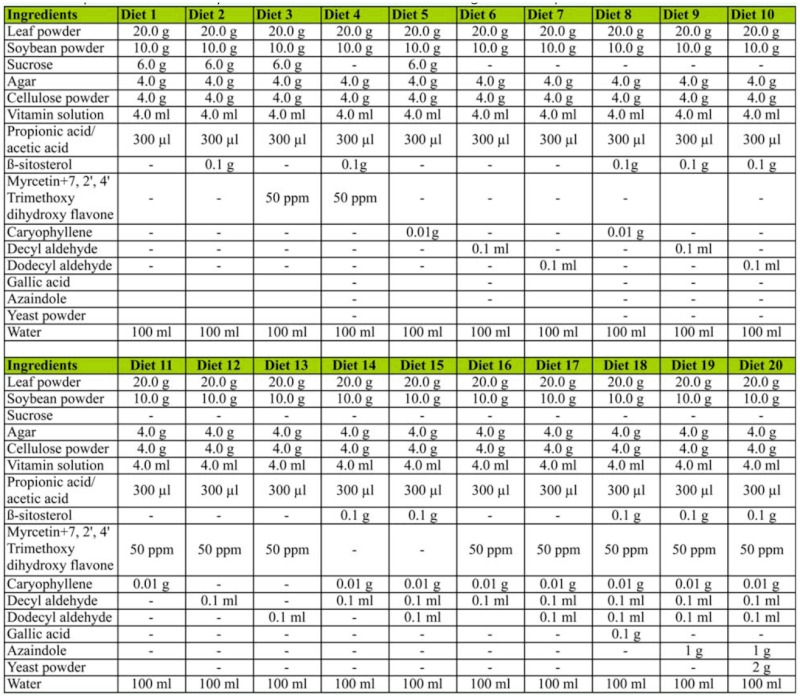
Composition of the semi-synthetic diet used for attraction and biting behavior experiments.

**Table 2.**
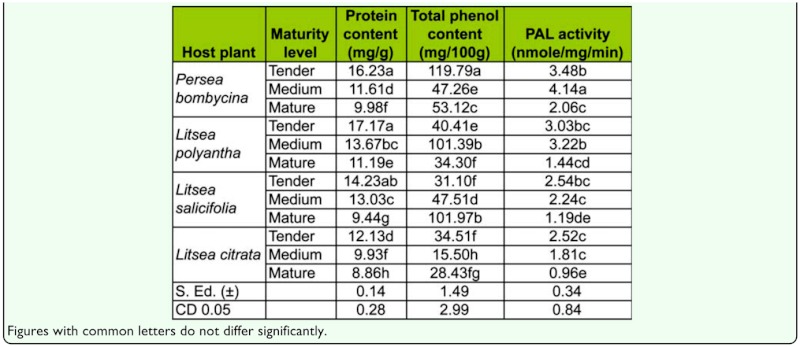
Variation of biochemical parameters with respect to tender, medium and mature leaves of different host plants.

**Table 3.**
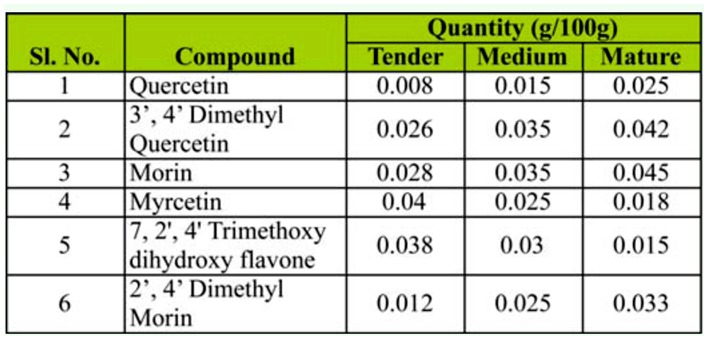
Comparative abundance of the phenolic compounds in the leaves of different age group of *Persea bombycina*.

**Table 4.**
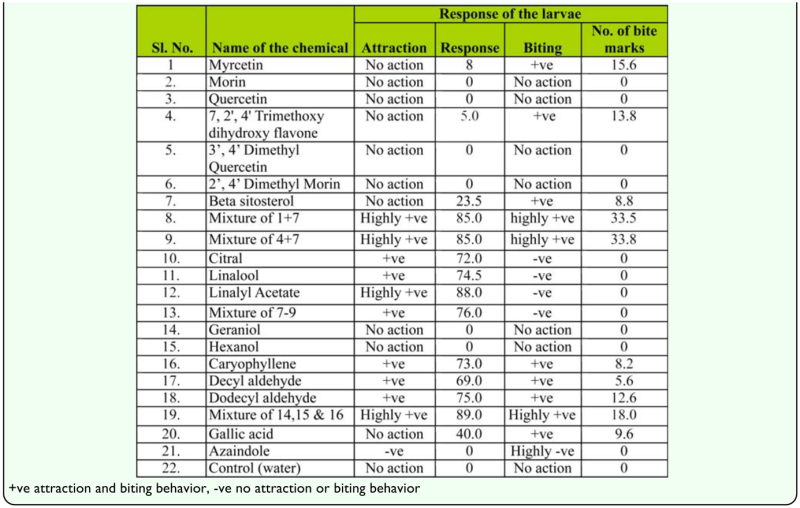
Attraction and biting response of the muga silkworm towards different chemicals

**Table 5.**
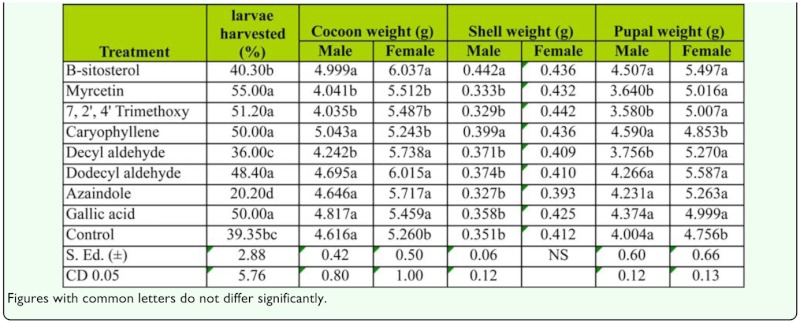
Rearing performance of muga silkworms fed with leaves treated with selected chemicals.

## Abbreviation:

**PAL**,: phenylalanine ammonia lyase
